# Agreement Between Exhaled Breath Carbon Monoxide Threshold Levels and Self-Reported Cigarette Smoking in a Sample of Male Adolescents in Jordan

**DOI:** 10.3390/ijerph120100841

**Published:** 2015-01-15

**Authors:** Nihaya Al-Sheyab, Khalid A Kheirallah, Linda J Thomson Mangnall, Robyn Gallagher

**Affiliations:** 1 Faculty of Nursing, Jordan University of Science and Technology, P.O. Box 3030, Irbid 22110, Jordan; 2 Faculty of Health, University of Technology, Sydney 2007, Australia; E-Mail: robyn.gallagher@sydney.edu.au; 3 Charles Perkins Centre, Faculty of Nursing and Midwifery, the University of Sydney, Sydney 2007, Australia; E-Mail: Linda.tm@sah.org.au; 4 Department of Public Health, Faculty of Medicine, Jordan University of Science and Technology, Irbid 22110, Jordan; E-Mail: Kkheiral@gmail.com; 5 Cardiothoracic, Surgical and Medical Telemetry Unit, Sydney Adventist Hospital, Sydney 2007, Australia

**Keywords:** cigarette smoking, exhaled Breath Carbon Monoxide, ROC, adolescent, Jordan, waterpipe use

## Abstract

This study aimed to measure the percent agreement between Exhaled Breath Carbon Monoxide (eBCO) measure using a piCO^+^ smokerlyzer^®^ and self-reported cigarette smoking status and to determine the optimal thresholds for definite identification of cigarette smokers of male school students in Jordan. A descriptive, cross sectional, study of a random sample of male adolescents in grades 7 and 8 from four public high schools in Irbid, completed an adaptation of a standardized Arabic-language tobacco smoking questionnaire and an eBCO measure. Sensitivity and specificity of the eBCO were calculated against self-reported cigarette smoking. Participants (*n* = 439) had a mean age of 12.5 years (SD = 0.50) and 174 (39.9%) reported being an ever smoker of whom 59 (33.9%) reported being a recent (30-day) smoker. The optimal eBCO cut-off point for recent smoking was 4.5 ppm with a sensitivity of 84.7% and specificity of 65.5%. Overall, eBCO can accurately identify recent smokers and distinguish them from non-smokers. The eBCO use enables healthcare professionals and researchers to assess efficacy of smoking cessation and prevention programs without necessarily relying on self-report. Further research is indicated to validate our findings and should be expanded to include females, detailed characteristics of cigarette and waterpipe smoking.

## 1. Introduction

Despite the decline in tobacco consumption in Western societies, it remains a serious public health concern in the Eastern Mediterranean Region (EMR), especially among adolescents [1,2]. The most susceptible age groups are those transitioning from middle to high school (grades 7 and 8). Any tobacco use among adolescents is concerning because early tobacco use increases the likelihood of chronic illness and premature death [3,4]. An alarming increase in the rate of adolescents’ waterpipe smoking (WPS), a major tobacco consumption method in the EMR [2,5,6], suggests that the current public health focus on cigarette smoking is vastly under-estimating the rates of tobacco consumption. Recent evidence also suggests that dual (WPS and cigarette) smoking rates are increasing amongst adolescents [2,5,6]. Today, adolescents’ WPS usage rates are more prevalent than cigarette smoking alone and dual use rates are on the rise among both genders [2,5,6]. The need for public health level tobacco use interventions is well established for youth in the in the EMR. However, failure to include WPS usage when measuring tobacco-use prevalence rates or evaluating the efficacy and effectiveness of tobacco use cessation interventions [7] may be confounding evaluations.

Tobacco smoking status is defined in terms of recent, regular, current, and ever. Multiple methods are used to assess tobacco smoking status. Self-reported smoking status has traditionally been the standard approach to distinguish smokers from non-smokers and to determine the level of adherence to smoking cessation initiatives. This has primarily been due to low costs, ease of implementation, and previous reports of high sensitivity and specificity [8,9,10]. Nonetheless, concerns have been raised with self-reported smoking status in vulnerable groups including adolescents [11]. Whilst this is often situational fear of judgment, the risk of disapproval from the evaluator to the adolescent may prevent disclosure. There is some evidence suggesting that adolescents tend to report ever smoking rather than risk censure by admitting to recent cigarette smoking [12]. It is likely this may be more pronounced within school settings where smoking may be a prohibited activity, thus it is probable that in some situations, self-report will under-estimate adolescent smoking rates [12,13]. However, self-reported smoking in survey studies has been shown to be valid in adolescents when confidentiality is assured, especially among males who tend to tell the truth about their smoking status as compared to females in Jordan, where cigarette use is a taboo. Accordingly, non-invasive biochemical measures have increasingly been used to determine the smoking status of adolescents [14]. These include portable devices such as the exhaled Breath Carbon Monoxide (eBCO) device [15,16,17]. The accuracy and validity of these methods are becoming increasingly important in monitoring responses to tobacco intervention programs [7]. The primary value of eBCO devices lies in determining recent tobacco smoking [18] with the half-life of eBCO in the vicinity of five hours, with some minor variations arising with age, gender, and physical activity [19].

The characteristics of eBCO devices and threshold levels to assess smoking status have never been investigated, nor validated, in the EMR where WPS is prevalent or dual smoking established [5,6]. To date, there has been no evaluation of biomarker assessment among Jordanian adolescents, especially males, who in particular report higher rates of smoking [6]. This study was conducted to assess the percent agreement between the eBCO using the piCO^+^ smokerlyzer^®^ and the self-reported cigarette smoking status in a random sample of male school adolescents in Jordan. Because the eBCO method has never been validated in this population before, sensitivity and specificity of the eBCO were calculated against self-reported cigarette smoking, which is the most common method to assess tobacco smoking status in Jordan.

## 2. Experimental Section

The methods and design have been described in detail elsewhere [20], but are summarized below.

### 2.1. Design

A cross sectional study design was used within the school setting and a random sampling technique used to select participants.

### 2.2. Setting and Sample

The study population included young adolescent, male public high school students from Irbid city, Northern Jordan. The study site was selected because previous studies had identified a high prevalence of smoking in this district [6,21,22,23]. Four schools were randomly selected, using an opaque envelope technique, from a list of all public high schools for males (*n* = 21) in Irbid city. Combined, the selected schools had a total population of 882 in grades 7 and 8 (number per school was 120, 307, 215 and 240 male students). All students in the 7th and 8th grades in the selected schools were eligible to participate in the study. After stratifying by school and grade, a simple random sampling procedure was used to randomly select a sample of students from each grade in each school using an opaque envelope technique. The number of randomly selected students in each school was proportionate to the enrolment size in that school.

### 2.3. Data Collection

A self-administered Arabic-language, smoking questionnaire, adapted from the Global Youth Tobacco Survey (GYTS) [24], and previously tested in Jordan [20,22] was used. This questionnaire is described in detail elsewhere [20] but included information regarding demographics (age, residency) and tobacco use (WPS and cigarettes), smoking status and frequency. Specifically, ten questions were taken from the GYTS. Five of these questions addressed the pattern of students’ active smoking behavior and included two “yes/no” questions about the use of cigarette and waterpipe smoking, one about the frequency of cigarette smoking (number of days smoked) in the past month, ranging from none to all days of the month, one about the total number of cigarettes per day (intensity) in the past month ranging from none to more than 20 cigarettes/day, and one about the age of initiation of cigarette smoking (ranging from ≤eight years to 13–14 years old).

#### 2.3.1. Smoking Classification

Students who reported that they had smoked cigarettes in the preceding 24 h were identified as recent cigarette smokers and adolescents who reported smoking at least once in their life were classified as ever cigarette smokers. Students who reported smoking cigarettes at least once during the week prior to data collection were identified as regular smokers whereas those who reported to have smoked cigarettes at least once in the month prior to data collection were identified as current smokers. Students who reported to have ever smoked waterpipe were identified as ever waterpipe users. Unlike cigarette smoking, WPS is not a behavior that school children can readily engage in before school or in the playground thus we have only used ever smoking for WPS to obtain an indication in this age group.

#### 2.3.2. eBCO Analyser

There are a number of portable, relatively inexpensive, eBCO devices available on the market and for this study, the piCO^+^ smokerlyzer^®^ (Bedfont Scientific, England, UK; measures in parts-per-million; ppm) was utilized. The piCO^+^ smokerlyzer^®^ was used in this study to detect eBCO levels with standard smoking threshold points provided by the manufacturer for adolescent and adult age categories. The standard cut-off points for adolescent smokers are: 0–4 ppm for a non-smoker, 5–6 ppm for students in the danger zone (light or casual smoker), 7–10 ppm for a smoker, 11–15 ppm for frequent smokers, and 16–25 for addicted smokers. The piCO^+^ smokerlyzer^®^ was initially calibrated by Bedfont Scientific [25] and was re-calibrated again during data collection as an additional measure to ensure accuracy of results. The piCO^+^ smokerlyzer^®^ is relatively cheap, is easy to use, and has been validated in tobacco control and prevention interventions in other countries [26]. The piCO^+^ smokerlyzer^®^ has an accuracy purely a measure of expired carbon monoxide, the ratio of eBCO is likely to be the same across inhaled tobacco products [19]. After finishing a cigarette the eBCO in expired air falls rapidly out to five minutes, then slows over the subsequent hour. eBCO was considered to have higher capacity in distinguishing cigarette smokers from non-smokers [27] and regular monitoring can lead to reduction of cigarette smoking [28]. In comparison to more invasive biochemical markers such as serum blood tests, eBCO has been demonstrated to show higher levels of accuracy [29,30].

### 2.4. Procedures

Utilizing the existing school system, all students were recruited in April 2013 with written parental consent prior to study commencement. Students were first assessed for their cigarette smoking and WPS behaviour using the self-administered questionnaire. The eBCO test was then attended by a trained research assistant and recorded on their questionnaire. All eBCO tests were conducted in the school hall between 10 am and 11 am. Data was collected in one day from each school and overall data collection was completed in one week.

#### eBCO Measurement Process

A breath sampling D-piece and a clean cardboard mouthpiece were attached to the piCO^+^ smokerlyzer^®^ before each student was tested. Students were asked to inhale and hold their breath whilst a 15-s countdown displayed. In order to empty lungs completely, at the end of the 15 s, students were coached to blow through the mouthpiece slowly and fully, as soon as the machine started beeping Students were informed of their test results immediately at the cessation of the test.

### 2.5. Statistical Analysis

Statistical Package for Social Sciences software application (SPSS, version 21, IBM Corp, Armonk, NY, USA) was used to analyze the data. Proportions and means (SD) were used to report categorical and continuous variables, respectively. The Independent samples t-test was used to compare mean eBCO levels against selected independent variables. Receiver-Operating Characteristic (ROC) curves were used to assess the optimal eBCO cut-off points that discriminate between recent smokers and non-recent smokers. Area Under the Curve (AUC) and standard error were reported to determine the ability of the piCO^+^ smokerlyzer^®^ to discriminate between recent smokers and non-smokers. Optimal eBCO cut-off points that provide the best balance between sensitivity and specificity were reported. Sensitivity and specificity were calculated by measuring the proportion of positive eBCO samples among self-reported recent smokers and the proportion of negative eBCO samples among non-smokers, respectively, using the optimal cut-off points. Sensitivity and specificity were also calculated for other types of cigarette smoking (regular, current, ever).

### 2.6. Ethical Considerations

Human Research Ethics Approvals were obtained from all institutions; Jordan University of Science and Technology and the Ministry of Education in Irbid district. Informed consent was obtained from the selected schools’ principals, initiated with a telephone call followed by signed written consent. Informed parental written consent was sought and obtained before the study commencement in addition to the individual students also being provided with a detailed age-appropriate study explanation, time of consideration and discussion with their parents and then asked for written consent.

## 3. Results

Of the 528 students invited to participate 452 (85.6%) were recruited, with the remaining 76 (14.4%) invited but not enrolled (31 (5.7%) were absent on data collection dates and 45 (8.5%) did not return the signed parental consent form) so 439 student results were included in the validation analyses (13 more were not included due to missing data). The mean age was 12.5 (SD = 0.50) years with participation almost equally distributed between grades 7 (48.2%) and 8 (51.8%).

Students’ tobacco use characteristics are presented in Table 1. Ever cigarette smoking and ever WPS prevalence rates were 39.9% and 53.5%, respectively. One third of all students (27.1%) reported being dual smokers (cigarettes and WPS). More than two thirds of students (69.6%) had at least one family member who was a cigarette smoker. A similar percentage of participants reported having their first cigarette by the time they were 14 years old, a third (27.3%) commencing at or before the age of 10 years and another third starting at the ages of 13 and 14 years (30.0%).

**Table 1:** Participants’ self-report smoking characteristics (*n* = 439).

Characteristic	Frequency	Percent
	*n*	%
Ever cigarette smoking	174	39.3
Ever WPS	232	53.5
**Ever Tobacco Use (WPS or Cigarettes)**		
Never	170	38.7
Cigarette only	43	9.8
WPS only	107	24.4
Dual (WPS & Cig)	119	27.1
Smoking Patterns among Ever Cigarette Smokers (*n* = 148) *		
Recent smokers **	59	39.9
Current smokers ***	111	75.0
Regular smokers ****	84	56.8

* Percentages may add up to more than 100%, 26 students reported ever cigarette smoking but not recent, current, or regular smoking; ** (today or last night); *** (≥once/last month); **** (≥once/last week).

Among those who self-reported as ever cigarette smokers (*n* = 148), more than one-third (39.9%) also reported recent cigarette smoking, totaling 13.4% of the total sample. Of ever smokers subgroup, three quarters (75.0%) were current cigarette smokers (smoked at least once in the last month), totaling 25.3% of all study students, and about half (56.8%) were regular cigarette smokers (smoked at least once in the last week) totaling 19.1% of the total sample. Overall, the mean (SD) eBCO level was 4.00 (SD = 2.01) ppm among all students whereas it was higher for cigarette smokers (M = 5.39, SD = 1.59).

A series of ROC validation analyses were performed on the four classifications of self-reported cigarette smoking (recent, regular, current, ever).

### 3.1. ROC Curve for Recent Cigarette Smokers

**Figure 1:**
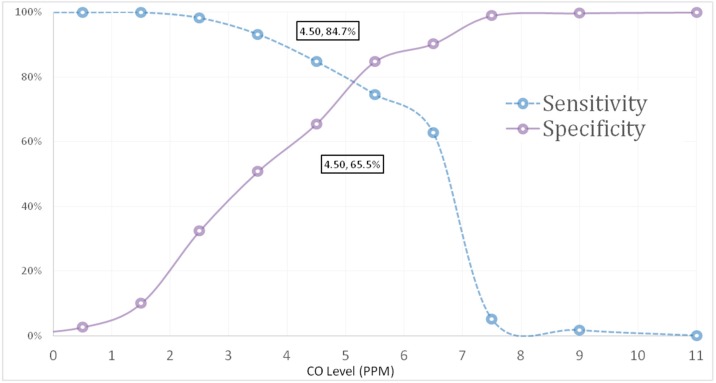
ROC curve for eBCO against self-reported recent cigarette smoking.

ROC analysis was performed against recent cigarette smoking (those who reported smoking on the day of data collection or the night before; *n* = 59)* versus* those who did not smoke cigarettes on the day or the night before data collection (*n* = 390). As illustrated in Figure 1, the AUC was 0.85 (SE = 0.025, *p* < 0.0001, 95% CI = 0.80–0.90) suggesting that eBCO test can significantly discriminate between participants in terms of recent smoking status.

An eBCO value of ≥4.5 ppm maximized the likelihood of accurately classifying recent smokers from non-recent smokers (Table 2). Although the highest total value of sensitivity and specificity (1.593) was at the 5.5 ppm cut off point, the sensitivity itself was 0.746, which is lower than that at 4.5 ppm threshold (Sensitivity = 0.847).

Using the 4.5 ppm eBCO value as a cut-off point, 131 non-recent smokers were incorrectly categorized as smokers (*i.e.*, False Positives (FP)), whereas only 9 smokers were incorrectly categorized as non-smokers (*i.e.*, False Negative (FN)). However, 249 non-smokers and 50 smokers were correctly categorized (*i.e.*, True Negatives (TN)) and True Positives (TP), respectively. This high proportion of FP reduces the PPV of the test. Nonetheless, the test returned good sensitivity at 4.5 ppm cut off point and was able to correctly detect recent smoking among those identified by self-reported as recent cigarette smokers. Similarly, eBCO test Positive Predictive Value (PPV) and the Negative Predictive Value (NPV), respectively, defined as the number of TP tests divided by the total number of positive tests, and the number of TN tests divided by the total number of negative tests, were 27.6% and 86.6%.

**Table 2:** Sensitivity and specificity of various cut-off levels of eBCO against recent and ever cigarette smoking.

Smoking status	Sensitivity & Specificity										
**Recent Smokers**											
Label	−1	0.5	1.50	2.50	3.50	4.50	5.50	6.50	7.50	9.00	11.00
Sensitivity	1.00	1.00	1.00	0.983	0.932	0.847	0.746	0.627	0.051	0.017	0.000
Specificity	0.00	0.026	0.10	0.324	0.508	0.655	0.847	0.903	0.989	0.997	1.00
Sensitivity + Specificity	1.00	1.026	1.10	1.307	1.44	1.502	1.593	1.53	1.04	1.014	1.00
1-specificity	1.00	0.974	0.900	0.676	0.492	0.345	0.153	0.097	0.011	0.003	0.000
**Ever Smokers**											
Sensitivity	1.00	1.00	1.00	0.948	0.851	0.747	0.466	0.333	0.029	0.006	0.000
Specificity	0.00	0.038	0.145	0.435	0.641	0.805	0.920	0.939	0.992	0.996	1.00
Sensitivity + Specificity	1.00	1.038	1.145	1.383	1.492	1.552	1.385	1.272	1.021	1.002	1.00
1-specificity	1.00	0.962	0.855	0.565	0.359	0.195	0.080	0.061	0.008	0.004	0.000

### 3.2. ROC Curve for Regular and Current Cigarette Smokers

For regular cigarette smoking, the AUC was 0.793 (SE = 0.027, *p* < 0.0001, 95% CI = 0.74–0.85) suggesting that the eBCO test also performed well in discriminating between regular smokers and non-smokers at 4.5 ppm cut off level (Sensitivity = 0.762, Specificity = 0.67), although the curve was better against recent smoking status (Figure 1). For current cigarette smoking, the AUC was 0.782 (SE = 0.024, *p* < 0.0001, 95% CI = 0.73–0.83) at 4.5 ppm cut off point (Sensitivity = 0.757, Specificity = 0.704) suggesting that the eBCO test also performed well in discriminating between current smokers and non-smokers, although the curve was better against recent smoking.

### 3.3. ROC Curve for Ever Cigarette Smokers

**Figure 2:**
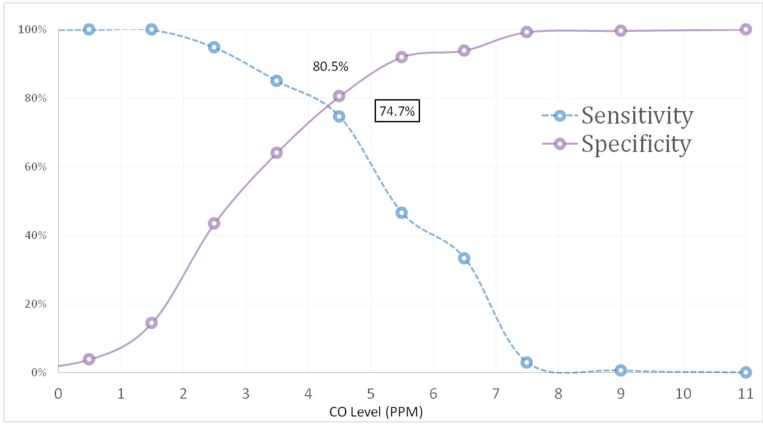
Sensitivity and specificity for various cut-off levels for eBCO against self-report of ever cigarette smoking.

Using self-report of ever cigarette smoking as the diagnostic test (Figure 2) the diagnostic accuracy of eBCO performed well in discriminating between smokers and non-smokers; AUC (SE) = 0.831 (SE = 0.20), *p*-value = 0.000 (95% CI = 0.79–0.87). This is an unexpected finding given the short half-life of carbon monoxide in lungs and is strongly indicative that some adolescents who reported ever cigarette smoking have smoked (or inhaled) tobacco more recently but did not disclose.

Sensitivity and specificity proportions for the eBCO are shown in Table 2. The optimal cut-off point of 4.50 ppm maximized the likelihood of accurately distinguishing ever smokers from non-smokers (Sensitivity = 0.747, Specificity = 0.805). Further, both sensitivity and specificity curves intersect at a cut-off level of 4.5 ppm resulting in the highest combined sensitivity and specificity value; Sensitivity + Specificity = 1.552 (Figure 2).

As illustrated in Table 3, a ≥ 4.5 ppm eBCO value maximized the likelihood of accurately classifying ever smokers from non-smokers (Sensitivity + Specificity = 1.552). Using this criterion as a cut-off, 51 non-smokers were incorrectly categorized as positive* i.e.*, FP, and 44 smokers were incorrectly categorized as non-smokers,* i.e.*, FN. However, 211 non-smokers and 130 smokers were correctly categorized;* i.e.*, TN and TP, respectively (Table 3). Similarly, eBCO test PPV and NPV, respectively, defined as the number of TPs tests divided by the total number of positive tests, and the number of true negative tests divided by the total number of negative tests, were 71.8% and 82.7%.

**Table 3:** Distribution of Students by eBCO Cut-off Point (4.5 ppm) and Self-Report Recent and Ever Cigarette Smoking *n* (%).

eBCO Smoking Status Threshold **	Self-Reported Recent Cigarette Smoking			Self-Reported Ever Cigarette Smoking		
	Recent	Non-Recent	Total	Ever	Non-Ever	Total
Smoker (≥4.5)	50 (27.6) TP *	131 (72.4) FP *	181 (41.2)	130 (74.7) TP	51 (19.5) FP	181(41.5)
Non-smoker (<4.5)	9 (0.04) FN *	249 (96.5) TN *	258 (59.2)	44 (25.3) FN	211 (80.5) TN	255 (58.5)
Total	59 (100.0)	380 (100.0)	439 (100.0)	174 (100.0)	262 (100.0)	436 (100.0)

** Smoking status as determined by the ROC curve cut-off point of ≥4.5; * TP: True Positive, FN: False Negative, FP: False Positive, TN: True Negative.

A statistically significant difference (*p* < 0.0001) in the mean eBCO levels among ever smokers (5.39, SD = 1.59) and non-smokers (3.09, SD = 1.73) was detected using 4.5 ppm eBCO level as the cut-off vale to discriminate between ever smokers and non-smokers.

## 4. Discussion

The study results demonstrate that low-cost eBCO devices may be used to accurately determine smoking in male adolescents and that these devices offer a sound alternative to self-report in dual smoking populations [30]. The eBCO, using the piCO^+^ smokerlyzer^®^, was a valid measure for discriminating smokers, (ever, current, regular and recent) from non-smokers* versus* self-report. The eBCO cut-off value of 4.5 ppm accurately classified current and ever smokers from non-smokers, however, the eBCO threshold of less than 4 ppm may optimally validate 24-h cessation and reduce misclassification of smokers as “abstinent” [31].

This study is unique in many ways and, primarily, to the authors’ knowledge, this is the first validation of a biomarker test among youth in the Middle Eastern region where the uptake of tobacco products is high. Additionally, eBCO levels were examined in a vulnerable age group—males in early adolescence (aged between 12 and 13 years old)—that has been poorly investigated. Also, the context of the study was a population with alarmingly high smoking rates in both adolescents [22,32] and adults [1] and where dual smoking occurs at higher rates than usual [20]. In this unique context, the eBCO could distinguish not only recent smoking, but also, regular, current, and ever smoking, which was surprising given that the piCO^+^ smokerlyzer^®^ is marketed as being most sensitive to recent smoking because of the expired carbon monoxide half-life of approximately 5 h. Specifically, our findings suggest that eBCO can accurately identify recent smokers and distinguish them from non-smokers, and this is congruent with previous literature that used different eBCO devices [27,31,33,34].

Our findings showed that adolescents who only reported ever cigarette smoking were also identifiable as their levels of carbon monoxide were equivalent to the recent smokers zone. This means that the test has good sensitivity and was able to properly detect recent smoking status among those who reported to actually be recent smokers, but our findings show that the False Positive (FP) rate is high (72.4%), meaning that the eBCO tested positive while the student self-reported to be a non-smoker. These findings are congruent with previous studies in pregnant women who had high levels of biochemical markers despite their report of being non-smokers [35]. A potential explanation is that self-reports are under-estimations as documented in a recent systematic review, which noted that smoking prevalence is both underestimated by self-report and that underestimation also varies by the population being studied [13]. This may be the case in the current study as male adolescents have previously been identified as have lower levels of accuracy when self-reporting recent cigarette smoking status [11]. Youth find it easier to report ever smoking but they are usually reluctant to confess to current and recent cigarette smoking [12].The social unacceptability of cigarettes has been addressed before but not through biochemical markers.

Other factors may also be important, including the dual use of both cigarettes and waterpipe in this population. WPS could have inflated the FP rates of cigarette smoking in our study. About half of the sample reported WPS, which, although unlikely, could have affected eBCO levels and thresholds if smoked recently. This assumption is particularly relevant as a recent local study reported much higher rates of dual use than that of cigarette smoking alone [5,32]. This might lead us to conclude that even if adolescents who reported ever cigarette smoking were telling the truth about cigarette smoking, they might have smoked waterpipe recently thus the device classified them as recent cigarette smokers. This would be worth further investigation.

Finally, a recent review of the literature highlighted the importance of the medium by which biological samples are measured [13]. Gorber* et al.* noted that when cotinine in body fluids is compared with self-reported smoking, misclassification for recent smokers, who self-reported as non-smokers occurred regularly across the 10 large studies (total *n* = 14,554) included in the total sample [16]. This finding contradicts previous research showing that the eBCO is highly unlikely to detect carbon monoxide in nonsmokers, especially among those who have not recently smoked and those who use low levels of tobacco [35]. It is perplexing that, for ever smoked, the piCO^+^ smokalyzer^®^ performed better in the regular and current smoking groups against self-report despite the fact that eBCO can only detect recent smoking due to its short half-life [36]. This finding makes it challenging to understand the extent of error in self-reported smoking prevalence amongst male adolescents such as the population examined in the current study.

Our findings also showed if the aim of measuring tobacco use is to test the effect of smoking cessation programs a lower cut-off point is needed to ensure sensitivity. We found that when self-reported recent smoking status is used as a reference tool a cut-off ≤4.5 ppm maximized sensitivity and specificity and the threshold required was 4.5 ppm, which is similar to the cut-off points recommended in the piCO^+^ smokerlyzer^®^ for adolescents. However, these cut off points are higher than that reported by Cropsey* et al.* [37] but similar to more recent evidence from two 2010 studies investigating the accuracy of the piCO^+^ meter with these authors recommending an abstinence cut off of ≤4 ppm [27,34].

### 4.1. Limitations

Limitations of the study should be noted including the possibility of inaccurate self-report as discussed. Study results may not be generalizable to the general population as the sample was urban dwelling male adolescents. Although all students were informed and encouraged to exhale slowly but deeply during measurement, exhalation time was not carefully monitored in this study, and might have influenced thresholds [34]. Pragmatically, it is difficult to coach breathing techniques to students until they can do it precisely. However, all researchers were thoroughly trained in the protocol of assessing eBCO levels. Lastly, the specificity of eBCO as a biomarker of cigarette smoking is limited by environmental sources of eBCO such as that from pollution and motor vehicle exhaust [33], however, these factors were not taken able to be factored into this study. The limitations are balanced by the study strengths which include a large random sample and a high response rate.

### 4.2. Recommendations

Future research needs to test students’ carbon monoxide breath after WPS, optimally during weekends or school holidays, where waterpipe usage is more accessible and probable, in order to assess how eBCO thresholds perform in a setting where cigarette use is contaminated by WPS. It would be worthwhile conducting a further study investigating female adolescents to compare findings. Additionally, in light of the high rates of dual smokers across both genders [5,32], adolescent smokers should be stratified in future research by cigarette only, WPS only, and dual-users as well. This may allow us to understand the expected error in self-reporting smoking prevalence among various subgroups of adolescents. Moreover, detailed characteristics of WPS including last time used, intensity and frequency, should be assessed in future research allowing precise measurement of eBCO levels for adolescents who use waterpipe regularly. The applicability of using the eBCO as a valid tool for assessing adolescent recent smoking status should be encouraged especially in smoking cessation and prevention programs. Finally, future research needs to assess the accuracy of self-report against the eBCO method among Jordanian adolescents.

## 5. Conclusions

The findings of our study are promising and suggest that eBCO levels using the piCO^+^ smokerlyzer^®^ can be an accurate, cost-effective method to identify smokers from non-smokers if careful and thorough assessment of tobacco use including recent smoking, second hand smoking, and WP use, is undertaken. This will allow us to assess efficacy of smoking cessation and prevention programs delivered by researchers and all healthcare professionals, especially in the school setting, among this vulnerable population. Finally, the eBCO method could be a more accurate tool when assessing recent tobacco exposure as compared to self-report.
